# Interaction of Human Dopa Decarboxylase with L-Dopa: Spectroscopic and Kinetic Studies as a Function of pH

**DOI:** 10.1155/2013/161456

**Published:** 2013-05-26

**Authors:** Riccardo Montioli, Barbara Cellini, Mirco Dindo, Elisa Oppici, Carla Borri Voltattorni

**Affiliations:** Section of Biological Chemistry, Department of Life Sciences and Reproduction, University of Verona, Strada Le Grazie 8, 37134 Verona, Italy

## Abstract

Human Dopa decarboxylase (hDDC), a pyridoxal 5′-phosphate (PLP) enzyme, displays maxima at 420 and 335 nm and emits fluorescence at 384 and 504 nm upon excitation at 335 nm and at 504 nm when excited at 420 nm. Absorbance and fluorescence titrations of hDDC-bound coenzyme identify a single pK_spec_ of ~7.2. This pK_spec_ could not represent the ionization of a functional group on the Schiff base but that of an enzymic residue governing the equilibrium between the low- and the high-pH forms of the internal aldimine. During the reaction of hDDC with L-Dopa, monitored by stopped-flow spectrophotometry, a 420 nm band attributed to the 4′-N-protonated external aldimine first appears, and its decrease parallels the emergence of a 390 nm peak, assigned to the 4′-N-unprotonated external aldimine. The pH profile of the spectral change at 390 nm displays a pK of 6.4, a value similar to that (~6.3) observed in both *k*_cat_ and *k*_cat_/K_*m*_ profiles. This suggests that this pK represents the ESH^+^ → ES catalytic step. The assignment of the pKs of 7.9 and 8.3 observed on the basic side of *k*_cat_ and the PLP binding affinity profiles, respectively, is also analyzed and discussed.

## 1. Introduction

Dopa decarboxylase (DDC; EC 4.1.1.28) is a pyridoxal 5′-phosphate- (PLP-) dependent enzyme which catalyzes the irreversible decarboxylation of L-Dopa and L-5-hydroxytryptophan (5-HTP), thus producing the neurotransmitters dopamine and serotonin. The enzyme accepts other catechol- or indole-related L-amino acids and has been therefore also named L-aromatic amino acid decarboxylase (AADC). Like other PLP enzymes [1–7], DDC is of clinical interest since it is involved either in Parkinson's disease, a degenerative disorder of the central nervous system resulting from the death of dopamine-generating cells in the *substantia nigra,* or in AADC deficiency, a rare inherited neurometabolic disease due to mutations on the *AADC *gene leading to deficit of catecholamines and serotonin in the central nervous system and periphery [8]. Thus, the elucidation of the structural and functional features of the enzyme is relevant for the development of treatment strategies for both disorders. In this regard, a structure-based design search aimed at identifying inhibitors of peripheral DDC more selective than those currently administered to patients with Parkinson's disease has been recently reported [9]. Moreover, the molecular basis of AADC deficiency analyzed by comparing the characteristics of normal human DDC (hDDC) with those of some pathogenic variants in their recombinant purified form has allowed not only to unravel their molecular defects but also to suggest appropriate therapeutic treatments for patients bearing the examined mutations [10, 11].

Progress in understanding the structure/function relationships operating in DDC has been obtained by means of kinetic, spectroscopic, and structural studies on the pig kidney and rat liver enzymes, either in the naturally occurring [12–17] or in the recombinant purified [18–26] forms, as well as, more recently, on recombinant purified hDDC [9–11, 27]. The crystal structure of pig kidney holo DDC alone and in complex with carbidopa (a substrate analog endowed with a hydrazinic group) has been solved at 2.6 and 2.5 Å resolutions, respectively [28]. The overall structure of the protein is a tightly associated dimer in which the active site is buried in the central part. Each monomer is composed of a large domain and a C-terminal domain, typical of the aspartate aminotransferase family (Fold Type I), as well as an N-terminal domain characteristic of Group II decarboxylases. Several other features of DDC are evident in these structures: (i) the way in which PLP is anchored to the enzyme involving His302 and His192, two highly conserved residues in *α*-decarboxylases [29]; (ii) how the inhibitor binds; and (iii) which amino acid residues might be involved in the catalytic activity. Unexpectedly, the crystal structure of hDDC in the apo form reveals that it exists in an open conformation in which the dimer subunits move 20 Å apart and the two active sites become solvent exposed. Moreover, by varying the PLP concentration in the crystals of the open DDC, two more structures have been solved, thus allowing to identify the structural determinants of the conformational change occurring upon PLP binding [27].

Although DDC enzymes share similar absorption spectra, that is, absorption maxima at 420 and 335 nm, pig kidney and rat liver enzymes display different PLP emission fluorescent properties, possibly due to the presence, even if to a different degree, of a species absorbing at 335 nm and emitting fluorescence at 390 nm in their apoenzyme forms [15, 24, 25]. These findings, together with the fact that the coenzyme absorbing bands show a modest pH dependence, do not allow to unequivocally assign the 335 nm absorbing band to a form of the internal aldimine and to understand how the equilibrium between the 420 and 335 nm species is governed. Moreover, although previous spectroscopic analyses of the reaction of both pig kidney and rat liver DDC with L-Dopa provided evidence for the appearance of two intermediates absorbing at 420 and 385 nm, the assignment of these species is conflicting. These absorbance bands have been attributed to 1-N-protonated-4′-N-protonated and 1-N-protonated-4′-N-unprotonated Schiff bases shown by the low- and high-pH forms of the external aldimine [13, 17]. On the other hand, other authors suggested that these species were formed during the course of the decarboxylation reaction, being the 420 nm and the 385 nm either the adsorption complex and the external aldimine with L-Dopa, respectively [25, 26], or two different external aldimines [30].

The present study presents a detailed investigation of the pH dependence of the bound coenzyme absorbance and fluorescence features and of the steady-state kinetic parameters of hDDC. Additionally, changes of the absorbance bands of hDDC upon L-Dopa binding as a function of pH have been monitored by rapid scanning stopped-flow experiments. Taken together, the results allow us to identify three observable ionizations in hDDC and to propose their involvement in the structures of the bound coenzyme and in the intermediates along the decarboxylation pathway.

## 2. Material and Methods

### 2.1. Chemicals

L-Dopa, PLP, 2,4,6-trinitrobenzene-1-sulfonic acid, isopropyl-*β*-D-thiogalactopyranoside, and protease inhibitor cocktail were purchased from Sigma-Aldrich. Bis-Tris-propane at a final concentration of 50 mM was used over the pH range 6–8.8. The other chemicals were of the highest purity available.

### 2.2. Enzyme Preparation

The conditions used for expression and purification of hDDC were as previously described [11]. The apo form was prepared as previously reported [11]. 

### 2.3. Binding Affinity of hDDC for PLP

The equilibrium dissociation constant for PLP, K_D(PLP)_, was determined by measuring the quenching of the intrinsic fluorescence of the apoenzyme (0.15 *μ*M) in the presence of PLP at a concentration range of 0.01–20 *μ*M. The experiments were carried out in 50 mM Bis-Tris-propane in the pH range 6–8.8. The K_D(PLP)_ values were obtained by fitting the data to the following equation:

(1) Y=YMAX[E]t+[PLP]t+KD(PLP)−([E]t+[PLP]t+KD(PLP))2−4[E]t[PLP]t2[E]t,

where [*E*]_*t*_ and [PLP]_*t*_ represent the total concentrations of hDDC dimer and PLP, respectively, *Y* refers to the intrinsic fluorescence quenching changes at a PLP concentration, [PLP], and *Y*_max⁡_ refers to the aforementioned changes when all enzyme molecules are complexed with the coenzyme.

### 2.4. Enzyme Assay

The decarboxylase activity toward L-Dopa was measured by the spectrophotometric assay described by Sherald et al. [31], and it was modified by Charteris and John [32]. Measurements were performed in the presence of 10 *μ*M PLP. Data of enzymatic activity as a function of substrate concentration were fitted to Michaelis-Menten equation.

### 2.5. Spectral Measurements

Absorption spectra were made with a Jasco V-550 spectrophotometer at a protein concentration of 10 *μ*M. Fluorescence spectra were taken with an FP750 Jasco spectrofluorometer using 5 nm excitation and emission bandwidths at a protein concentration of 10 *μ*M. The enzyme solution was drawn through a 0.2 *μ*m filter to reduce light scattering from the small amount of precipitate. Spectra of blanks, that is, samples containing all components except hDDC, were taken immediately before the measurements of samples containing protein.

### 2.6. pH Studies

Absorbance and fluorescence data were fitted to (2) and (3):

(2) $$\begin{array}{c} A=\frac{A_{1}-A_{2}}{1+10^{\text{pH}-\text{p}{\text{K}}_{\text{spec}}}}+A_{2}, \end{array}$$

(3) $$\begin{array}{c} A=\frac{A_{1}-A_{2}}{1+10^{\text{p}{\text{K}}_{\text{spec}}-\text{pH}}}+A_{2}, \end{array}$$

where *A*_1_ and *A*_2_ are the higher and the lower absorbance limits at a particular wavelength, respectively.

The log K_D(PLP)_, log *k*_cat_, log *k*_cat_/K_*m*_, and values for hDDC versus pH were fitted to the following appropriate equations:

(4) $$\begin{array}{c} \log Y=\log\frac{Y_{L}+Y_{H}\left(H/{\text{K}}_{1}\right)}{1+H/{\text{K}}_{1}}, \end{array}$$

(5) $$\begin{array}{c} \log Y=\log\frac{C}{1+H/{\text{K}}_{A}+{\text{K}}_{B}/H}, \end{array}$$

(6) $$\begin{array}{c} \log Y=\log\frac{C}{1+H/{\text{K}}_{A}}, \end{array}$$

where K_*A*_ and K_*B*_ represent the ionization constants for enzyme or reactant functional groups, *Y* is the value of the parameter observed as a function of pH, *C* is the pH-independent value of *Y*,  *H* is the hydrogen ion concentration, and *Y*_*L*_ and *Y*_*H*_ are constant values at low and high pH, respectively. 

### 2.7. Pre-Steady-State Kinetic Analysis by UV-Vis Stopped-Flow Spectroscopy

hDDC was mixed using a Biologic SFM300 mixer with an equal volume of 2 mM L-Dopa in 50 mM Bis-Tris-propane at pH ranging from 6.0 to 8.0. Coenzyme absorbance changes were monitored with a TC-100 (path length of 1 cm) quartz cell coupled to a Biokine PMS-60 instrument. The dead time was 3.8 ms at a flow velocity of 8 mL/sec. Absorbance scans (800) from 300 to 600 nm were collected on a logarithmic time scale with a J&M Tidas 16256 diode array detector (Molecular kinetics, Pullman, WA). Data were analyzed using either SPECFIT (Spectrum Software, Claix, France) or Biokine 4.01 (Biologic, Claix, France) to determine the observed rate constants. Single-wavelength time courses were fit to an equation of the following general form:

(7) $$\begin{array}{c} A_{t}=A_{\infty }\pm \sum A_{i}\text{ex}{\text{p}}^{\left(-k_{\text{obs}}t\right)}, \end{array}$$

where *A*_*t*_ is the absorbance at time *t*, *A*_*i*_ is the amplitude of each phase, *k*_obs_ is the observed rate constant for each phase, and *A*_*∞*_ is the final absorbance.

## 3. Results

### 3.1. pH Dependence of the Internal Aldimine and Coenzyme Binding Affinity

**Figure 1:**
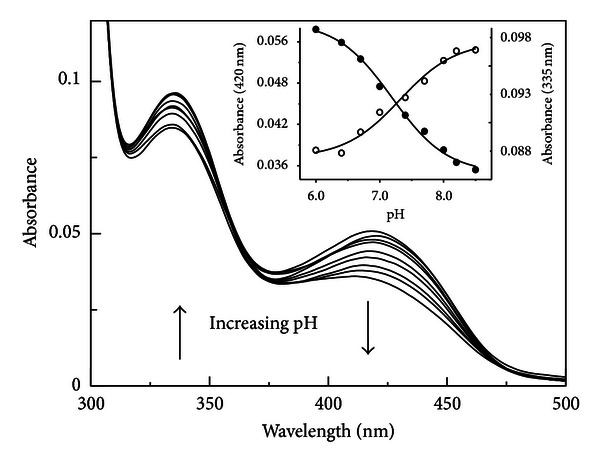
pH dependence of the visible spectra of hDDC. Absorbance spectra of 10 *μ*M hDDC were acquired in 50 mM Bis-Tris-propane at pH 6.0, 6.4, 7.0, 7.4, 7.7, 8.0, 8.2, and 8.5. The inset shows the pH dependence of the absorbance at 420 (•) and 335 nm (∘). The solid lines represent the theoretical curves according to (2) and (3).

Apo hDDC completely lacks absorbance in the visible region, while the holo form is characterized by absorbance bands at 420 and 335 nm associated with positive dichroic signals at the same wavelengths [11]. The absorbance spectrum of hDDC in the holo form changes as a function of pH over the range 6–8.5 (Figure 1): the 335 nm band increases with pH, while the 420 nm band decreases. The spectra do not show a clear isosbestic point, thus suggesting the presence of multiple species. When we fitted the data to curves with one, two, or three ionizations, we found that they fit best to a model with one ionization ((2) and (3)): the pK_spec_ values obtained were 7.2 ± 0.1 and 7.3 ± 0.1 for the absorbance at 420 and 335 nm, respectively (Figure 1, inset) (Table 1).

**Figure 2:**
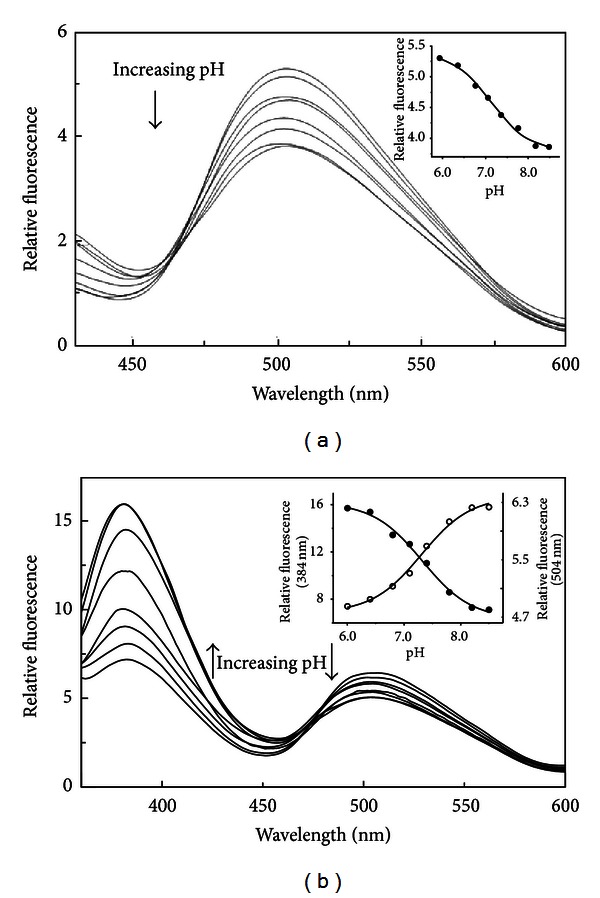
pH dependence of the internal aldimine emission fluorescence of hDDC. Emission fluorescence spectra of 10 *μ*M hDDC in 50 mM Bis-Tris-propane at pH 6.0, 6.4, 6.8, 7.1, 7.4, 7.8, 8.2, and 8.5 upon excitation at 420 nm (a) and 335 nm (b). The inset of (a) shows the pH dependence of the emission intensity at 504 nm (•), while that of (b) shows the pH dependence of the emission intensity at 504 nm (∘) and 384 nm (•). The solid lines represent the theoretical curves according to (2) and (3).

Excitation of hDDC at 420 nm results in an emission band at 504 nm, whose intensity decreases as pH increases below a single pK of 7.2 ± 0.1 (Figure 2(a) and inset). Moreover, hDDC emits at 384 and 504 nm upon excitation at 335 nm. Emission fluorescence intensity at 384 nm increases with increasing pH, while that at 504 nm decreases (Figure 2(b)). As shown in the inset of Figure 2(b), the pH profile for the 384 nm emission intensity increases above a single pK of 7.3 ± 0.1, while that at 504 nm decreases below a single pK of 7.1 ± 0.1 (Table 1). When emission was observed at 384 nm, the excitation spectrum exhibits a maximum at 337 nm, whereas at 500 nm, the excitation spectrum displays maxima at 341 and 410 nm.

**Figure 3:**
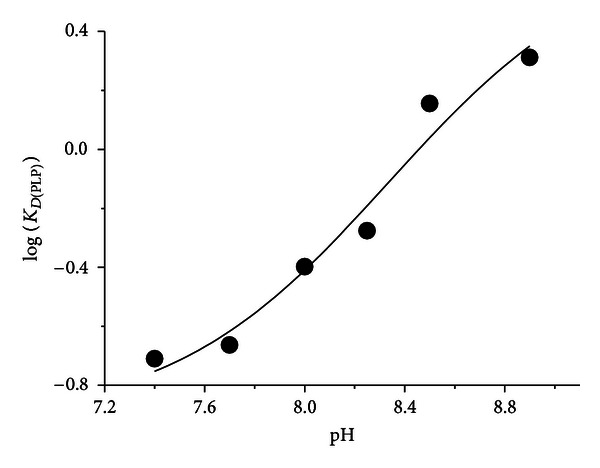
pH dependence of K_D(PLP)_ of hDDC. K_D(PLP)_ values were determined in 50 mM Bis-Tris-propane at the indicated pH as described under “Section 2”. The points shown are the experimental values (expressed in *μ*M), while the curve is from fit to the data using (4).

The K_D(PLP)_ value for hDDC at pH 7.4 is 0.2 *μ*M, and it increases as the pH increases. The pH dependence of K_D(PLP)_ fits well (4), giving a pK_D(PLP)_ value of 8.3 ± 0.2 (Figure 3, Table 1). 

### 3.2. pH Dependence of Kinetic Parameters for hDDC

**Figure 4:**
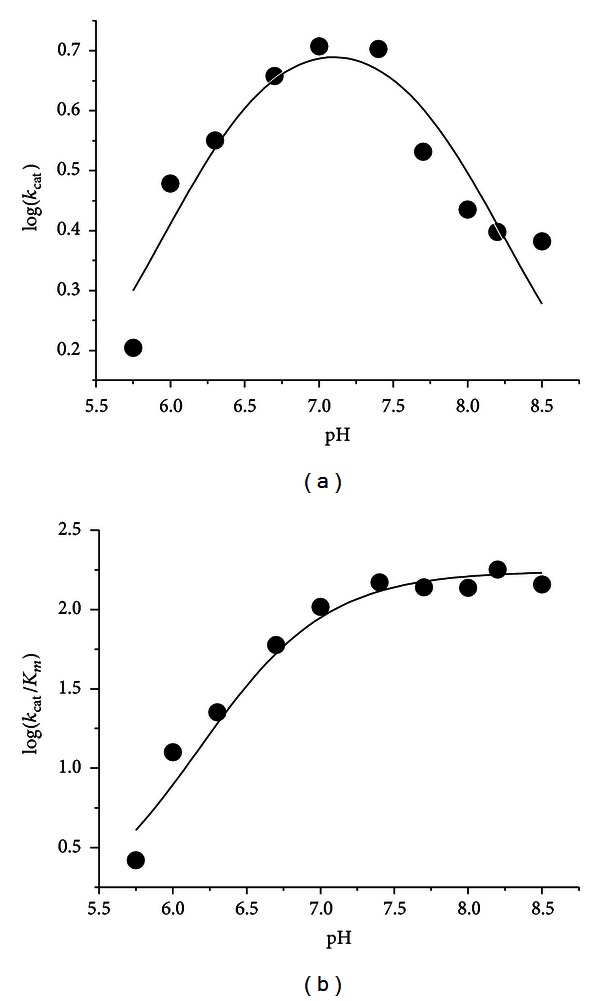
pH dependence of the kinetic parameters for the decarboxylase activity of hDDC toward L-Dopa. (a) Log *k*_cat_ profile and (b) log *k*_cat_/K_*m*_ profile. The assays were performed at 25°C in 50 mM Bis-Tris-propane at the indicated pH using 50 nM enzyme concentration in the presence of 10 *μ*M exogenous PLP. The points shown are the experimental values, while the curves are from fits to the data using (5) for log *k*_cat_ and (6) for log *k*_cat_/K_*m*_.

The pH dependence of the kinetic parameters for hDDC toward L-Dopa was determined, and the results are shown in Figures 4(a) and 4(b). The variation with pH of log *k*_cat_ gives rise to a bell-shaped profile: fitting the data to (5) and (6) yields pK values of 6.3 ± 0.1 and 7.9 ± 0.1; Log *k*_cat_/K_*m*_ decreases below a pK of 6.1 ± 0.1 (Table 1).

### 3.3. pH Dependence of External Aldimine with L-Dopa

**Figure 5:**
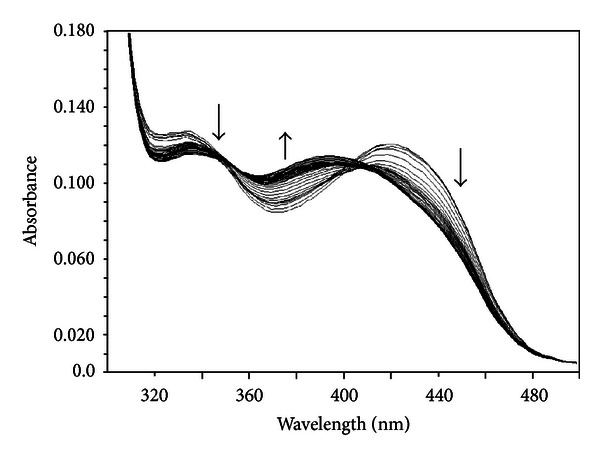
Time-resolved spectra for the reaction of hDDC with L-Dopa. Rapid scanning stopped-flow spectra obtained upon reaction of hDDC (20 *μ*M) with L-Dopa (2 mM) in Tris-Bis-propane, pH 7.4, at 25°C. Spectra were taken between 1 and 100 ms at 1 ms intervals and between 101 and 200 ms at 10 ms intervals.

**Figure 6:**
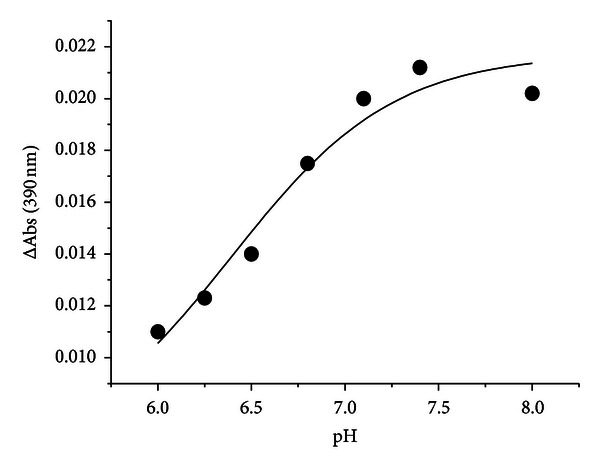
pH dependence of the amplitude of the change of the 390 nm absorbance band in the reaction of hDDC with L-Dopa. Amplitude of the 390 nm absorbance band monitored by rapid scanning stopped-flow spectra upon reaction of hDDC (20 *μ*M) and L-Dopa (2 mM) in Tris-Bis-propane, at 25°C at the indicated pH. The points shown are the experimental values, while the curve is from fit to the data using (3).

To obtain information about the identity of intermediates in the reaction of hDDC with L-Dopa we carried out rapid-kinetic spectroscopic studies over the pH range 6–8. Upon mixing the enzyme with L-Dopa at a saturating concentration, a biphasic spectral change was observed. In the first phase, a rapid increase in the absorbance at 420 nm and a decrease in the 335 nm band were observed within 50 ms, followed by a second phase, in which the absorbance at 420 nm decreases and concomitantly a new absorbance band appears at 390, occurring with a rate of 31 s^−1^ (Figure 5). The amplitude of the absorbance changes at 390 nm increases with pH above a single pK of 6.4 ± 0.3 (Figure 6) (Table 1). It should be also noted that at pH higher than 6.4 the appearance of the 390 nm band is accompanied by that of a shoulder absorbing at ~440 nm. Considering that the enzyme-dopamine complex absorbs at ~400 nm and that only a modest amount of dopamine (20 *μ*M) is formed at the end of the second phase, the shoulder cannot be attributed to the enzyme-dopamine complex. A likely attribution might be a quinonoid species, which, according to Metzler et al. [33], could absorb at wavelengths lower than 500 nm.

**Figure 7:**
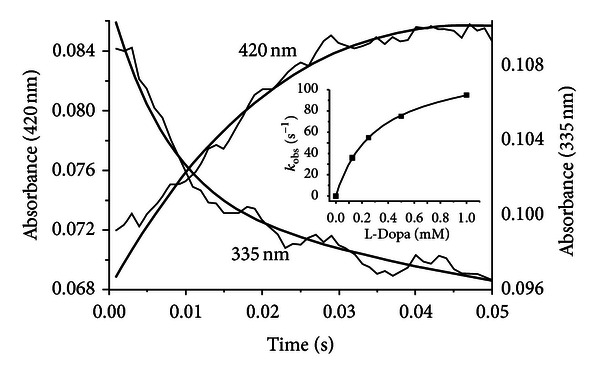
Single-wavelength stopped-flow measurements of the reaction of hDDC with L-Dopa at pH 6.0 and 15°C. Reaction of hDDC (20 *μ*M) with L-Dopa (1 mM) was carried out at 15°C in 50 mM Bis-Tris-propane (pH 6.0). Time courses at 420 and 335 nm are reported. The solid lines are from fits to (7). The inset shows the dependence of *k*_obs_ for the increase in the intensity of the 420 nm band as a function of the final concentrations of L-Dopa after mixing. The points shown are the experimental values, while the curve is from fit to the data using (8).

**Figure 8:**
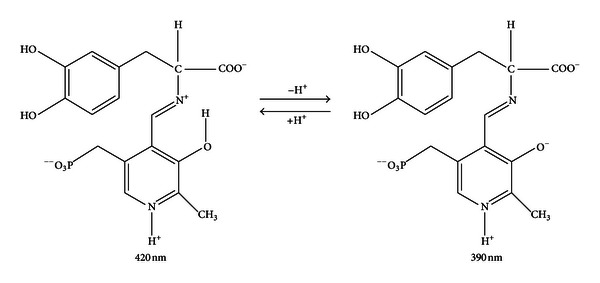
Structures of the coenzyme-L-Dopa complexes absorbing at 420 nm and 390 nm.

In order to establish if the intermediate absorbing at 420 nm represents a Michaelis complex or an external aldimine, its rate of formation as a function of L-Dopa concentration was measured. We decided to carry out these measurements at 15°C and at pH 6.0, that is, under experimental conditions in which the decarboxylation reaction is slow enough so that its kinetics can be measured. As shown in Figure 7, upon addition of L-Dopa to hDDC, an increase in the 420 nm band with a concomitant decrease in the 335 nm signal can be seen. The apparent first-order rate constant of the appearance of the 420 nm band, *k*_obs_, shows a hyperbolic dependence on L-Dopa concentration in the range 0.08–1 mM (inset of Figure 7). The *k*_obs_ data were fitted to the following equation:

(8) $$\begin{array}{c} k_{\text{obs}}=k_{+2}\frac{\left[\text{L-Dopa}\right]}{{\text{K}}_{1}+\left[\text{L-dopa}\right]}+k_{-2}, \end{array}$$

which describes the following two-step binding model assuming that the first step is rapid:

(9) E+L-Dopa⇄K1E-L-Dopa  intermediate ⇄k−2k+2E-L-Dopa  Schiff  base,

where K_1_ is the dissociation constant for the intermediate (Michaelis complex or geminal diamine) formed prior to the formation of the Schiff base species, and *k*_+2_ and *k*_−2_ are first-order rate constants for an interconversion between the intermediate and the Schiff base. Estimated values of *k*_+2_ and K_1_ based on the data in the inset of Figure 7 are 124 ± 3 s^−1^ and 0.32 ± 0.02 mM, respectively, while the *k*_−2_ value is nearly zero. The K_1_ value is consistent with the K_*m*_ value at pH 6.0 (0.23 ± 0.03 mM) measured under steady-state conditions, thus strongly suggesting that the intermediate absorbing at 420 nm represents a 1-N-4′-N-protonated external aldimine. It follows that the rate of formation of the Schiff base at 25°C can be estimated to be around 250 s^−1^ using the empirical rule of a 2-fold reduction for a 10°C reduction in temperature. This value is considerably higher (~80-fold) than the *k*_cat_ value at 25°C at pH 6.0 (3 s^−1^), thus indicating that one of the catalytic steps after the external Schiff base formation, including the decarboxylation step, is rate determining in the entire catalytic reaction [34]. All together, these data indicate the consecutive formation of two external aldimines, one absorbing at 420 nm and the other at 390 nm, whose structures are presented in Figure 8. 

## 4. Discussion

The pH dependence of catalysis and spectral features has been studied in details for only a few PLP-dependent enzymes [35–39]. This allowed to elucidate how ionizations control their activities. Up to date, such analyses have been hampered for DDC for the following reasons: (i) a large portion of the coenzyme covalently bound is present in both pig kidney and rat liver enzymes in an inactive form and shows absorbance and PLP emission fluorescence similar to those of the corresponding holoenzymes and (ii) both pig kidney and rat liver DDC enzymes show little absorbance changes with altering pH. 

Unlike the apo form of pig kidney and rat liver DDC, the apo hDDC does not display any absorbance in the 330 nm region and does not exhibit PLP emission fluorescence. Thus, we decided to perform a detailed investigation of the pH dependence of the spectroscopic properties of hDDC in its internal and external aldimine forms, of the PLP binding affinity as well as of the steady-state kinetic parameters. The following discussion attempts to assign the pK values observed in these analyses. 

**Scheme 1:**
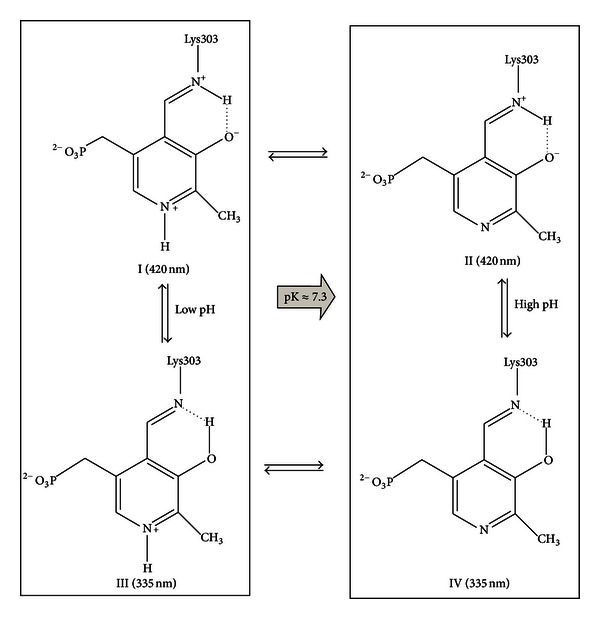
Putative model for the pH dependence of the internal aldimine.

The titration of hDDC-bound coenzyme absorbance and fluorescence over the pH range 6–8.5 is consistent with a deprotonation event with a pKa value of ~7.3, which could be the result of the deprotonation of the internal aldimine. However, there is no red shift in the 335 nm band at pH values higher than the apparent pK, as would be expected for the unprotonated aldimine absorbing at 360 nm. Structures which could account for an increase in the 330 nm region at high pH have been postulated to arise either from the formation of an adduct upon addition of a deprotonated nucleophilic or a hydroxyl group to the imine double bond or from the conversion from the ketoenamine to the enolimine tautomer. The attribution of the 335 nm absorbance band to a substituted aldamine is in contrast with the following data: (i) when hDDC is treated with NH_2_OH, the absorbance bands at 420 and 335 nm are completely lost, and the resultant apoenzyme lacks the PLP emission fluorescence, (ii) binding of L-Dopa causes the disappearance of the 335 absorbance band, (iii) upon excitation at 335 nm, the 384 nm fluorescence emission remains at low pH where the substituted aldamine would be destabilized by protonation, and (iv) the fluorescence excitation spectrum at the emission wavelength of 384 nm shows that the absorbance band that gives the excited state is seen at 341 nm, which is apparently longer than the wavelength generally observed for substituted aldamine structures, 330–335 nm. Thus, it should be taken in consideration the possibility that the 335 nm band could be attributed to the enolimine tautomer of the Schiff base. Both 384 and 504 nm emission maxima are seen upon excitation of hDDC at 335 nm, and acid promotes the 504 nm emission at the expense of the 384 nm emission. Honikel and Madsen have shown that the enolimine can emit either solely at ~400 or ~500 nm, or at a combination of both wavelengths, depending on the polarity and acidity of the solvent. Fluorescence emission at ~400 nm versus 500 nm is determined by a competition between (i) proton transfer from the enolimine structure at the excited state to the aldimine nitrogen of the ketoenamine in the singlet excited state and (ii) radiative decay of the excited to the ground state [40]. On the basis of all these considerations, we can conclude that the 384 nm fluorescence emission of hDDC results from the excited state of the enolimine tautomer of the Schiff base before it has tautomerized to the ketoenamine excited state. A similar explanation has been proposed for the pH-dependent spectral changes observed for dialkylglycine decarboxylase [37], serine glyoxylate aminotransferase [38], histidine decarboxylase [41], and glutamate decarboxylase [42]. Examination of the absorbance titration curves indicates that at pH values much lower than the apparent pK (~7.2) some 335 nm is still present and that at pH values higher than pK some 420 nm absorbing species remains. Thus, one might expect from our results that the pyridine nitrogen in hDDC is not fully protonated, as it is usually assumed for PLP enzymes having an aspartate residue interacting with the pyridine nitrogen. Accordingly, the model depicted in Scheme 1 is proposed: the N-protonated (I) and N-unprotonated (II) ketoenamine forms absorb at 420 nm, while the N-protonated (III) and N-unprotonated (IV) enolimine tautomers absorb at 335 nm. At pH values less than pK I and III will be present, while II and IV represent the forms at pH much higher than the pK. Ring nitrogen protonation state governs the two equilibria between I and II absorbing at 420 nm as well as between III and IV absorbing at 335 nm, that is, between species spectroscopically indistinguishable. Thus, the most likely explanation that accounts for the pH titration of coenzyme absorbance and fluorescence is that the ionization observed is not associated with any functional group on the Schiff base itself. Rather, it is an active site residue in close proximity to the coenzyme whose ionization alters the ratio between the two tautomers that absorb at 420 and 335 nm. 

The *k*_cat_ profile of the decarboxylation reaction catalyzed by hDDC toward L-Dopa displays two pKa values at about 6.3 and 7.9, thus suggesting that one group is required to be unprotonated and a second group protonated to achieve maximum velocity. The pKa on the acidic side of the profile is similar to that seen in the *k*_cat_/*K*_*m*_ profile, and, thus, it can be concluded that this group is involved in catalysis but not in binding. Since it has been proven that in pig kidney DDC CO_2_ release is rate limiting, *k*_cat_ must report on ionization(s) of the external aldimine. Thus, the spectral changes taking place in the bound coenzyme upon addition of L-Dopa have been analyzed as a function of pH by rapid scanning stopped-flow spectroscopy. The kinetic analyses of the reaction of hDDC with L-Dopa clearly demonstrate the presence of two intermediates absorbing at 420 and 390 nm. The 420 nm absorbing species is formed first, followed by formation of the second intermediate absorbing at 390 nm. The amplitude of the signal change at 390 nm increases with increasing pH above a single pK of ~6.4. The finding that the rate of appearance of the 420 nm band measured at pH 6.0 shows that a hyperbolic dependence on the concentration of L-Dopa is consistent with the assignment of this absorbance band to a 4′-N-protonated external aldimine. The time and pH dependence of the conversion of the 420 to the 390 nm absorbance band strongly suggest that the 390 nm band could be attributed to a 4′-N-unprotonated external aldimine. Even in the absence of the spectra of these intermediates, Minelli et al. [30] have predicted the presence of the step ESH^+^ → ES, corresponding to the 420 → 390 nm conversion, along the reaction pathway of the decarboxylation of L-Dopa. Our results validate this proposal, thus ruling out that the 420 nm intermediate represents Michaelis complex, as previously suggested [25, 26]. The pK of this spectral transition (6.4) roughly coincident with those observed in both *k*_cat_ and *k*_cat_/*K*_*m*_ profiles strengthens the argument that this ionization is associated with a catalytic event. 

There is no evidence to support an assignment of the pK (7.9) observed on the basic side of the *k*_cat_ profile to a specific group. It is likely the same one seen in the pH profile for *K*_D(PLP)_ (8.3). Its presence on the *K*_D(PLP)_ profile could suggest that the phosphate ester of the coenzyme phosphate group is the likely origin of this pK, which has a value similar to that observed for the coenzyme phosphate group in *Treponema denticola* cystalysin [35]. In hDDC the effect of this ionization could be the loss of the hydrogen bond between the hydroxyl group of Ser149 and the coenzyme phosphate group oxygen. Considering the large conformational change accompanying the transition from the apo to the holo form of hDDC [27], it can be speculated that the loss of hydrogen bond not only decreases the PLP binding affinity but also could hamper a correct apo → holo conversion resulting in a less catalytically competent conformation as the pH increases above 8. Nonetheless, it cannot be ruled out that the pK observed in *K*_D(PLP)_ does not coincide with the pK of the *k*_cat_ profile. In this case, the effect on the *k*_cat_ could be exerted by the ionization of a residue that, although remote from the active site, could affect the active site. A good candidate could be Tyr332 for which a role in C*α* protonation of the quinonoid along the decarboxylation pathway has been identified [22]. Although this assignment should be taken with caution, it is not in contrast with the detection at pH higher than pK of a ~440 nm absorbing shoulder attributable to a quinonoid species. Taken together, these results indicate that the maximum *k*_cat_ value of hDDC is achieved when the deprotonation of the external aldimine and that of an unidentified residue take place.

In the light of these data, the absence of a band absorbing at 390 nm in the reaction with L-Dopa of four DDC variants responsible for AADC deficiency, an inherited rare neurometabolic disease, should be reconsidered. In these variants, mutations concerning amino acid residues that interact directly or indirectly with PLP and/or its microenvironment, cause a perturbation of the active site [11]. It can be postulated that in these variants the external aldimine absorbing at 420 nm is not in a proper position and/or orientation to transfer the proton at 4′N of the Schiff base. Taking into account that, according to Minelli et al. [30], the 390 nm form is about 5-fold more reactive than the 420 nm one, it can be speculated that their reduced catalytic activity could be ascribable, at least in part, to the lack of the 420 → 390 nm conversion. 

## 5. Conclusions

A detailed investigation of the pH dependence of the steady-state kinetic parameters, of the spectroscopic titrations of the internal and external aldimine, as well as of the PLP binding affinity allows us to identify three observable ionizations in hDDC. The following tentative assignments for these have been made: pK1 (6.3-6.4), the deprotonation of the 4′-N-protonated external aldimine occurring during the decarboxylation pathway, pK2 (~7.2), a residue governing the equilibrium between the low- and the high-pH forms of the internal aldimine, and pK3 (7.9, 8.3), two distinct groups (the coenzyme phosphate ester of the internal aldimine and a residue involved in the catalysis) or a unique residue affecting both PLP binding affinity and *k*_cat_ value: additional studies will be needed to sort out the various possibilities.

## Figures and Tables

**Table 1:** Summary of pK_a_ values for hDDC.

Parameter	pH-independent value	pK_1_	pK_2_
*k*_cat_	5.1 ± 0.4 s^−1^	6.3 ± 0.1	7.9 ± 0.1
*k*_cat_/K_m_	174 ± 3 mM^−1^s^−1^	6.1 ± 0.1	
Absorbance at 420 nm pH titration			7.2 ± 0.1
Absorbance at 335 nm pH titration			7.3 ± 0.1
Emission fluorescence pH titration (exc. 335 nm)			
emis._384 nm_			7.3 ± 0.1
emis._504 nm_			7.2 ± 0.1
Emission fluorescence pH titration (exc. 420 nm)			
emis._504 nm_			7.2 ± 0.1
Amplitude ext. ald. 390 nm		6.4 ± 0.3	
K_D(PLP)_			8.3 ± 0.2

## Abbreviations

hDDC: Human Dopa decarboxylase
PLP: Pyridoxal 5′-phosphate
AADC: L-Aromatic amino acid decarboxylase
*K*_D(PLP)_: Equilibrium dissociation constant for PLP.
